# Clinical, Neurophysiological, and MRI Markers of Fampridine Responsiveness in Multiple Sclerosis—An Explorative Study

**DOI:** 10.3389/fneur.2021.758710

**Published:** 2021-10-26

**Authors:** Sepehr Mamoei, Henrik Boye Jensen, Andreas Kristian Pedersen, Mikkel Karl Emil Nygaard, Simon Fristed Eskildsen, Ulrik Dalgas, Egon Stenager

**Affiliations:** ^1^Department of Regional Health Research, University of Southern Denmark, Odense, Denmark; ^2^Department of Neurology, University Hospital of Southern Jutland, Sønderborg, Denmark; ^3^Open Patient Data Explorative Network, Odense, Denmark; ^4^Neurological Research Unit, MS Clinics of Southern Jutland (Sønderborg, Esbjerg, Kolding), University Hospital of Southern Jutland, Aabenraa, Denmark; ^5^Department of Brain and Nerve Diseases, University Hospital of Lillebælt, Kolding, Denmark; ^6^Department of Research and Learning, University Hospital of Southern Jutland, Aabenraa, Denmark; ^7^Center of Functionally Integrative Neuroscience (CFIN), Department of Clinical Medicine, Aarhus University, Aarhus, Denmark; ^8^Exercise Biology, Department of Public Health, Aarhus University, Aarhus, Denmark

**Keywords:** multiple sclerosis, performance test, magnetic resonance imaging, neurodegeneration, demyelination, neurophysiology

## Abstract

**Objective:** Persons with multiple sclerosis (PwMS), already established as responders or non-responders to Fampridine treatment, were compared in terms of disability measures, physical and cognitive performance tests, neurophysiology, and magnetic resonance imaging (MRI) outcomes in a 1-year explorative longitudinal study.

**Materials and Methods:** Data from a 1-year longitudinal study were analyzed. Examinations consisted of the timed 25-foot walk test (T25FW), six spot step test (SSST), nine-hole peg test (9-HPT), five times sit-to-stand test (5-STS), symbol digit modalities test (SDMT), transcranial magnetic stimulation (TMS) elicited motor evoked potentials (MEP) examining central motor conduction times (CMCT), peripheral motor conduction times (PMCT) and their amplitudes, electroneuronography (ENG) of the lower extremities, and brain structural MRI measures.

**Results:** Forty-one responders and eight non-responders to Fampridine treatment were examined. There were no intergroup differences except for the PMCT, where non-responders had prolonged conduction times compared to responders to Fampridine. Six spot step test was associated with CMCT throughout the study. After 1 year, CMCT was further prolonged and cortical MEP amplitudes decreased in both groups, while PMCT and ENG did not change. Throughout the study, CMCT was associated with the expanded disability status scale (EDSS) and 12-item multiple sclerosis walking scale (MSWS-12), while SDMT was associated with number of T2-weighted lesions, lesion load, and lesion load normalized to brain volume.

**Conclusions:** Peripheral motor conduction time is prolonged in non-responders to Fampridine when compared to responders. Transcranial magnetic stimulation-elicited MEPs and SDMT can be used as markers of disability progression and lesion activity visualized by MRI, respectively.

**Clinical Trial Registration:** www.ClinicalTrials.gov, identifier: NCT03401307.

## Introduction

Multiple sclerosis (MS) is a chronic inflammatory and demyelinating disease affecting myelinated axons in the central nervous system (CNS) and it is the leading cause of non-traumatic disability in young adults (1). Demyelination impairs neural conduction (2–5) leading to various motor, sensory, sphincter, visual, and cognitive symptoms among others (6).

**Figure 1:**
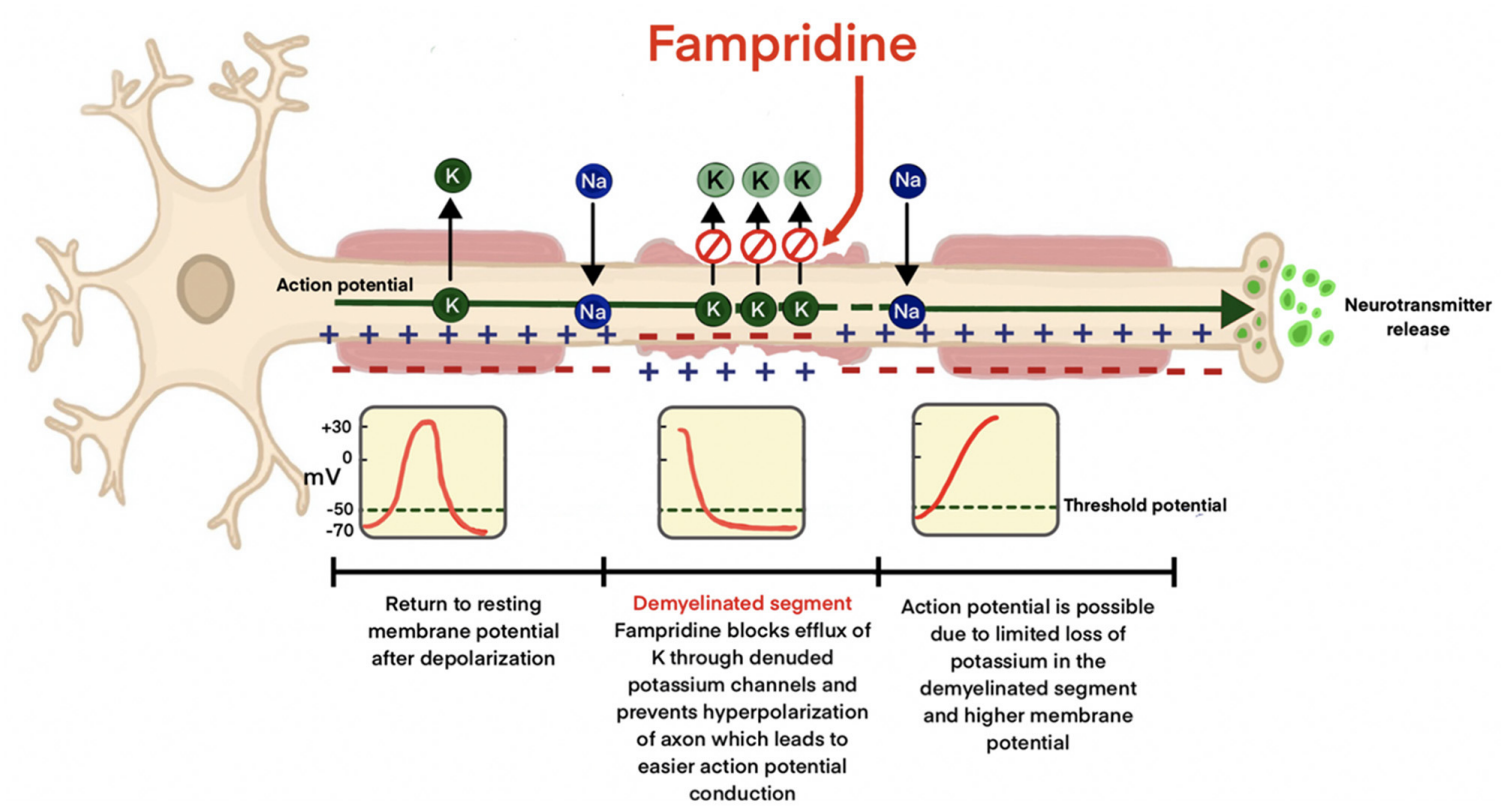
Mechanism of action of Fampridine in demyelinated axons. Potassium efflux from the voltage-gated potassium channels in the denuded axon is prevented, minimizing hyperpolarization leading to improved neural conduction.

Up to 75% of persons with multiple sclerosis (PwMS) are affected by walking impairments (7) which is considered by PwMS to be one of the most important bodily functions (4, 8, 9). Currently, Fampridine is the only pharmacological agent approved for the treatment of walking impairments in MS (10) and exerts its effect in both the CNS and the peripheral nervous system (PNS) (11). The primary mechanism of Fampridine is inhibition of potassium efflux from voltage-gated and other, yet unspecified (1), potassium channels from demyelinated axons (Figure 1) (1, 12). In addition, Fampridine has also shown beneficial effects on hand dexterity and cognitive processing speed (13).

In clinical trials, Goodman et al. estimated the proportion of PwMS responding to Fampridine treatment to be ~35–43% (14), while more recent studies suggest the proportion to be higher (15, 16). Responders are generally defined as PwMS improving by ≥20% in the timed 25-foot walk test (T25FW) when receiving 10 mg Fampridine twice daily in a 2-week trial (17). According to Danish national neurological treatment guidelines, Fampridine is only prescribed for PwMS who have walking disabilities and an expanded disability status scale (EDSS) 4–7 and who respond to Fampridine treatment in the treatment trial, whereas non-responders to Fampridine treatment are discontinued after a treatment trial with improvements <20% in the T25FW. The response to Fampridine treatment has been shown to be independent of MS subtype, disease duration, demographics, and type of disease modifying treatment (14, 18).

Currently, there are no established predictors for response to Fampridine treatment (7). However, pre-therapy central motor conduction times (CMCT) (19), baseline 6-min walking test (7), resting membrane threshold in transcranial magnetic stimulation (TMS) studies (10), and mean diffusivity and radial diffusivity in diffusion tensor imaging (DTI) have been suggested as biomarkers of Fampridine responsiveness (20).

To our knowledge, there are no longitudinal studies examining physical and cognitive performance tests, neurophysiological, magnetic resonance imaging (MRI) outcomes, and their associations in responders and non-responders to Fampridine treatment.

Consequently, the main objectives of this explorative study were to identify if biomarkers, evaluated in a cross-sectional study by Mamoei et al. (21), would remain stable at 1-year follow-up in PwMS with walking impairments, who already were established as responders and non-responders to Fampridine treatment. These biomarkers are applied in the evaluation of disability measures, physical and cognitive performance, neurophysiology, and brain MRI. As a further objective, associations between neurological disability measures, physical and cognitive performance, neurophysiology, and MRI measures were examined.

## Materials and Methods

**Figure 2:**
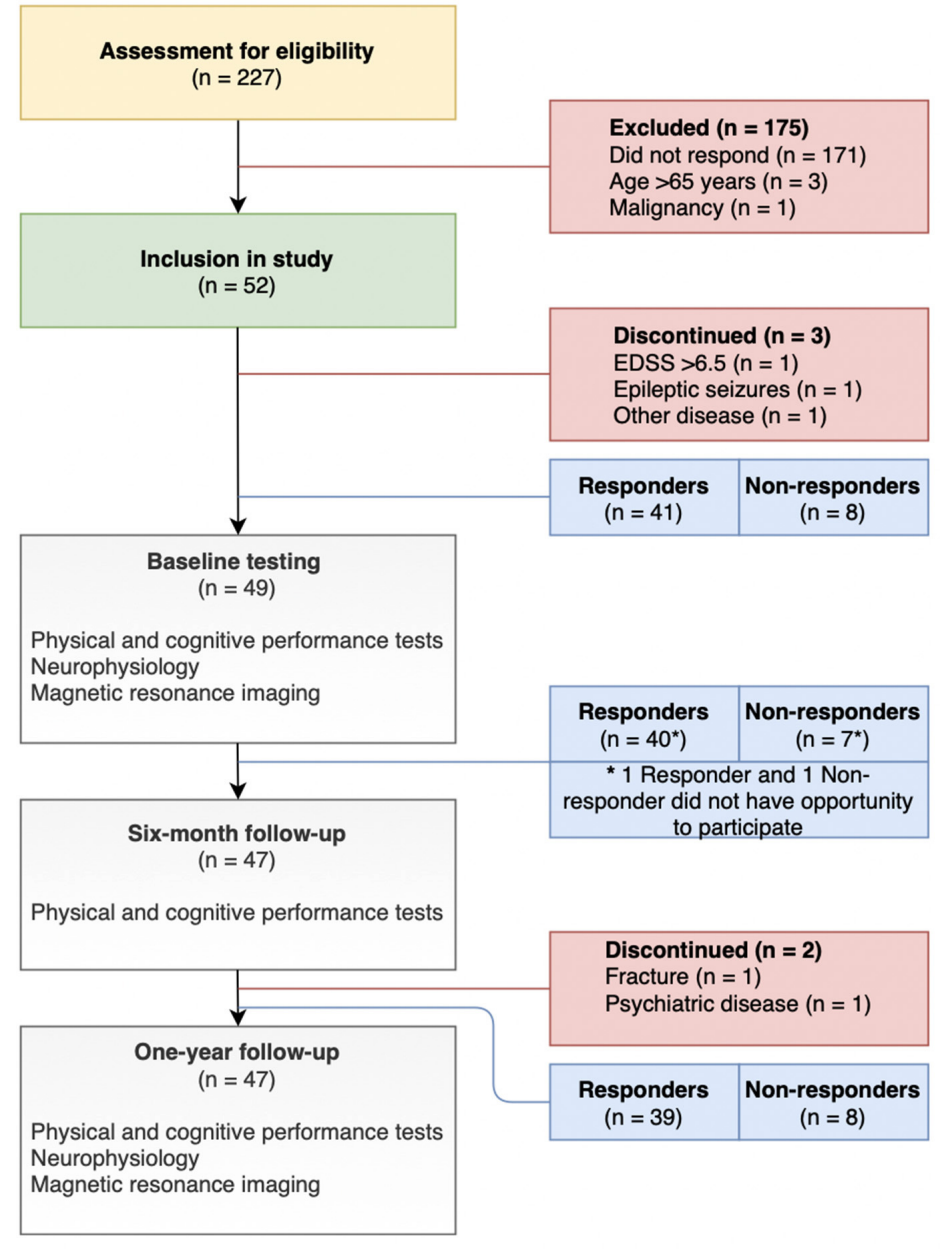
Flowchart of study design and participant selection.

This paper presents longitudinal data from an explorative prospective cohort study conducted from October 2018 to July 2020. PwMS, already established as responders and non-responders to Fampridine treatment, were examined at baseline and 1-year follow-up with physical and cognitive performance tests, neurophysiology, and MRI (Figure 2). At 6-month follow-up, only physical and cognitive performance tests were performed. Responders were defined as PwMS who had improved ≥20% on the T25FW after a 2-week trial receiving 10 mg Fampridine twice daily. Those with improvements below 20% were classified as non-responders. Accordingly, responders to Fampridine treatment in this study received treatment with 10 mg Fampridine twice daily as part of their usual symptomatic treatment in the MS-clinics. However, non-responders in this study did not receive Fampridine treatment following Danish neurological guidelines, as they did not improve ≥20% on the T25FW in the initial treatment trial outlined above.

### Subjects

#### Recruitment

Participants were assessed for eligibility, invited, and included via the MS-clinics of the Region of Southern Denmark (Odense, Kolding, Esbjerg, and Sønderborg) and a nationwide announcement from the Danish MS Society.

#### Inclusion and Exclusion Criteria

Inclusion criteria were MS diagnosis according to the McDonald criteria (22), an EDSS score below 7, responders to Fampridine treatment should be in active Fampridine therapy, non-responders to Fampridine treatment should not be in Fampridine therapy, and age between 18 and 65 years. Exclusion criteria were risk factors related to peripheral neuropathy (diabetes mellitus, impaired glucose tolerance, alcohol abuse, radiation treatment, and nutritional disorders), epileptic seizures, intracranial or intraocular metallic/foreign objects, pacemakers or implanted devices, and pregnancy.

### Examinations

#### Physical and Cognitive Performance Tests

Performance tests consisted of walking performance measured by T25FW (23) and six spot step test (SSST) (24), and lower body strength with the five-times sit-to-stand test (5-STS) (25). Manual dexterity was evaluated with the nine-hole peg test (9HPT) (26) and cognitive processing speed with the symbol digit modalities test (SDMT) (27).

#### Neurophysiological Examinations

Dantec Keypoint software and surface electromyography (EMG) was used when recording EMG responses of MEP and ENG examinations. Singe-pulse TMS was performed using a Dantec Magnetic Primer TwinTop & MagLite r-25 Magnetic Stimulator and a MagVenture MagPro R30 Transcranial Magnetic Stimulator. The primary motor cortex (M1) was stimulated using a handheld circular coil over the vertex. After determining the resting motor threshold (RMT), a stimulation intensity of 120% above the RMT was applied. Motor evoked potentials (MEPs) of the vastus medialis (VM) and tibialis anterior were elicited by TMS. Motor evoked potential signals to the VM and TA muscles were facilitated by asking the participants to do a slight voluntary knee extension and dorsiflexion of the ankle, respectively. Electroneuronography (ENG) of the peroneal and tibial nerves was performed using a bipolar surface electrode with pulse durations of 0.1 ms. Electrode placements and distances to the stimulation sites were undertaken using a ruler to ensure reproducibility with the methods applied in the baseline study (21).

Outcomes of TMS-elicited MEPs consisted of CMCT in the CNS, peripheral motor conduction times (PMCT) in the proximal part of the PNS, and their respective amplitudes. The motor conduction in the peripheral aspect of the PNS was evaluated with ENG of the tibial and peroneal nerves examining distal latencies, amplitudes, nerve conduction velocities (CV), and F-waves.

#### MRI Acquisition and Processing

Magnetic resonance imaging's were conducted in five hospitals in the Region of Southern Denmark. Scanners utilized were made by Siemens (Odense, Esbjerg, Sønderborg, and Aabenraa) and Phillips (Kolding) with field strengths of 1.5 Tesla (Odense, Kolding, Esbjerg, Sønderborg, and Aabenraa) and 3 Tesla (2 participants in Kolding). T2-FLAIR voxel sizes were 1.0 ×1.0 ×1.0 mm^3^ (Odense), 0.5 ×0.5 ×1.0 mm^3^ (Kolding), 0.5 ×0.5 ×2.5 mm^3^ (Esbjerg), and 0.7 ×0.7 ×6.5 mm^3^ (Sønderborg and Aabenraa). T1 voxel sizes were 1.0 ×1.0 ×1.0 mm^3^ (Odense), 0.7 ×0.7 ×6.5 mm^3^ (Esbjerg), and 0.9 ×0.9 ×1.0 mm^3^ (Sønderborg and Aabenraa). Follow-up MRI images were repeated at the same place as baseline for each participant.

Magnetic resonance imaging images were denoised (28) and corrected for bias field-induced intensity inhomogeneity (29), registered to the Montreal Neurological Institute space (30) and intensity normalized to the ICBM152 template (using T1 and T2 templates, respectively) (31). Brain extraction based on non-local segmentation technique (BEaST) were used to skull strip processed images (32). T2 hyperintense lesions were segmented using the Lesion Prediction Algorithm as implemented in the Lesion Segmentation Toolbox version 2.0.15 (https://www.statistical-modelling.de/lst.html) for SPM12 in MATLAB R2016b (MathWorks, Natick, MA).

Whole-brain volumes were estimated using skull stripped T2-FLAIR images. T1-weighted images were used for whole-brain estimation in cases, where these had higher resolutions than T2-FLAIR (*n* = 10).

Magnetic resonance imaging outcomes consisted of brain volume, number of T2-weighted lesions, volume of T2-weighted lesions (lesion load), and lesion load normalized to brain volume.

### Statistical Methods

To assess changes between baseline and 1-year longitudinal outcomes, descriptive statistics were utilized and stratified for responders and non-responders. Normality was assessed by Q-Q plots and histograms. Subsequently, paired *t*-tests or Wilcoxon sign rank tests were utilized, when comparing baseline and follow-up outcomes, depending on the data distribution. Levene's test was used to assess equal variances and Welch test was applied when the equal variance assumption was violated.

Mixed effect regression models were applied, as sequential measurements on participants were done. These models allow for analysis of associations across time points, while also minimizing biased estimates of treatment effect (33). Data analysis was based on univariate analysis, partly adjusted analysis examining for confounding, and fully adjusted analysis examining the effect of multicollinearity between exposure variables. Mixed effect regression models were adjusted for visit, age, and disease duration.

The mean value of right and left leg neurophysiological measures was analyzed as a reflection of the global affection of corticospinal pathways in MS, as suggested by Zeller et al. (19) and Brambilla et al. (20).

STATA 15.1 was used for statistical data analysis with *p*-values < 0.05 indicating statistical significance. Coefficients from regression models are reported with a 95% confidence interval (95% CI) and data in tables are presented as means ± standard deviations (SD).

### Standard Protocol Approvals

Approvals were gained from The National Committee on Health Research Ethics (project identification: S-20160204) and the Danish Data Protection Agency (Journal number: 16/42475). Clinicaltrials.gov identifier for this study is NCT03401307. The project was conducted in accordance with the Helsinki Declaration.

## Results

There were 41 responders and 8 non-responders to Fampridine treatment at baseline examinations, of which 39 and 8 participants (unmatched) completed the 1-year follow-up, respectively (Figure 2). Baseline characteristics were similar between groups regarding age, disease duration, gender, 12-item multiple sclerosis walking scale (MSWS-12), and EDSS (*p* > 0.05).

### MSWS-12, and Physical and Cognitive Performance Tests

At baseline and follow-up, there were no intergroup differences in disability measures and performance tests (*p* > 0.05). At 1-year follow-up (Table 1), responders to Fampridine treatment performed worse on the T25FW, SSST, 9-HPT, and SDMT compared to baseline values (*p* < 0.023). Non-responders performed worse only on the T25FW and the SDMT (*p* < 0.011).

**Table 1:** Baseline characteristics and changes in physical and cognitive performance at baseline and after 1 year in responders and non-responders to Fampridine treatment.

	**Responders**				**Non-responders**				**Intergroup difference**	
	**Baseline**	**Follow-up**	**Mean and**	***p*-Value**	**Baseline**	**Follow-up**	**Mean and**	***p*-Value**	**Baseline**	**Follow-up**
	**(*n* = 41)**	**(*n* = 39)**	**%-change**		**(*n* = 8)**	**(*n* = 8)**	**%-change**		***p*-value**	***p*-value**
Age; years	51.5 ± 8.2				50.1 ± 5.7				0.783^c^	
Disease duration; years	16.0 ± 7.0				18.5 ± 8.2				0.216^d^	
Gender; female/male	23/18				3/5				0.335^f^	
MSWS-12; *a.u*.	43.1 ± 10.4	43.5 ± 9.1	0.4 ± 6.8 (4.7 ± 27.6%)	0.327^a^	44.0 ± 11.8	45.0 ± 9.4	1.0 ± 11.4 (10.7 ± 46.9%)	0.810^a^	0.497^c^	0.670^c^
EDSS; *a.u*.	5.0 ± 1.3	5.1 ± 1.4	0.2 ± 0.5 (3.1 ± 10.4%)	0.103^b^	4.6 ± 1.3	4.8 ± 1.1	0.1 ± 0.2 (3.3 ± 6.2%)	0.346^b^	0.318^d^	0.226^d^
T25FW; *s*	8.5 ± 9.5	8.9 ± 10.4	0.4 ± 1.6 (6.6 ± 13.1%)	**0.003** ^**b**^	8.6 ± 6.0	11.2 ± 10.3	2.6 ± 4.3 (21.4 ± 16.4%)	**0.008** ^**b**^	0.665^d^	0.308^d^
SSST; *s*	14.5 ± 20.3	15.0 ± 19.5	0.4 ± 2.3 (6.1 ± 14.1%)	**0.023** ^**b**^	14.3 ± 8.9	18.4 ± 14.3	4.0 ± 5.6 (21.6 ± 20.8%)	0.055^a^	0.892^d^	0.224^d^
5-STS; *s*	11.9 ± 5.8	12.4 ± 6.6	0.5 ± 2.1 (5.0 ± 17.0%)	0.296^b^	14.1 ± 5.3	16.2 ± 7.9	2.1 ± 4.2 (12.2 ± 23.8%)	0.383^a^	0.457^d^	0.213^d^
9-HPT; *s*	27.5 ± 12.4	28.3 ± 13.7	1.6 ± 4.0 (4.6 ± 10.6%)	**0.015** ^**b**^	33.3 ± 9.8	37.0 ± 12.4	3.7 ± 5.0 (10.2 ± 15.3%)	0.074^a^	0.150^e^	0.104^e^
SDMT; *a.u*.	40.2 ±1 0.6	38.7 ± 9.6	−2.4 ± 6.1 (−4.7 ± 16.4%)	**0.020** ^**a**^	39.8 ± 5.8	34.1 ± 5.0	−5.6 ± 4.6 (−13.4 ± 12.0%)	**0.011** ^**a**^	0.920^c^	0.202^c^

*EDSS, expanded disability status scale; MSWS-12, 12-item multiple sclerosis walking scale; T25FW, timed 25-foot walk test; SSST, six spot step test; 5-STS, 5-times sit-to-stand test; 9-HPT, 9-hole peg test; SDMT, symbol digit modalities test; s, seconds; a.u., arbitrary units*.

*p-values derived from*

a *paired t-tests*,

b *Wilcoxon matched pairs signed rank test*,

c *unpaired t-tests*,

d * Wilcoxon rank-sum test*,

e *Bonferroni corrections for multiple testing, and*

f *chi-squared test. Bold p-values signify statistically significant differences*.

At 1-year follow-up, mean changes in non-responders to Fampridine treatment (Table 1) exceeded point estimates of the minimum clinically important differences (MCID) of the T25FW at 14.2% (10/39 responders and 4/8 non-responders) and SSST at 14.7% (7/39 responders and 5/8 non-responders) as suggested by Jensen et al. (34).

### TMS-Elicited MEPs and ENG

**Figure 3:**
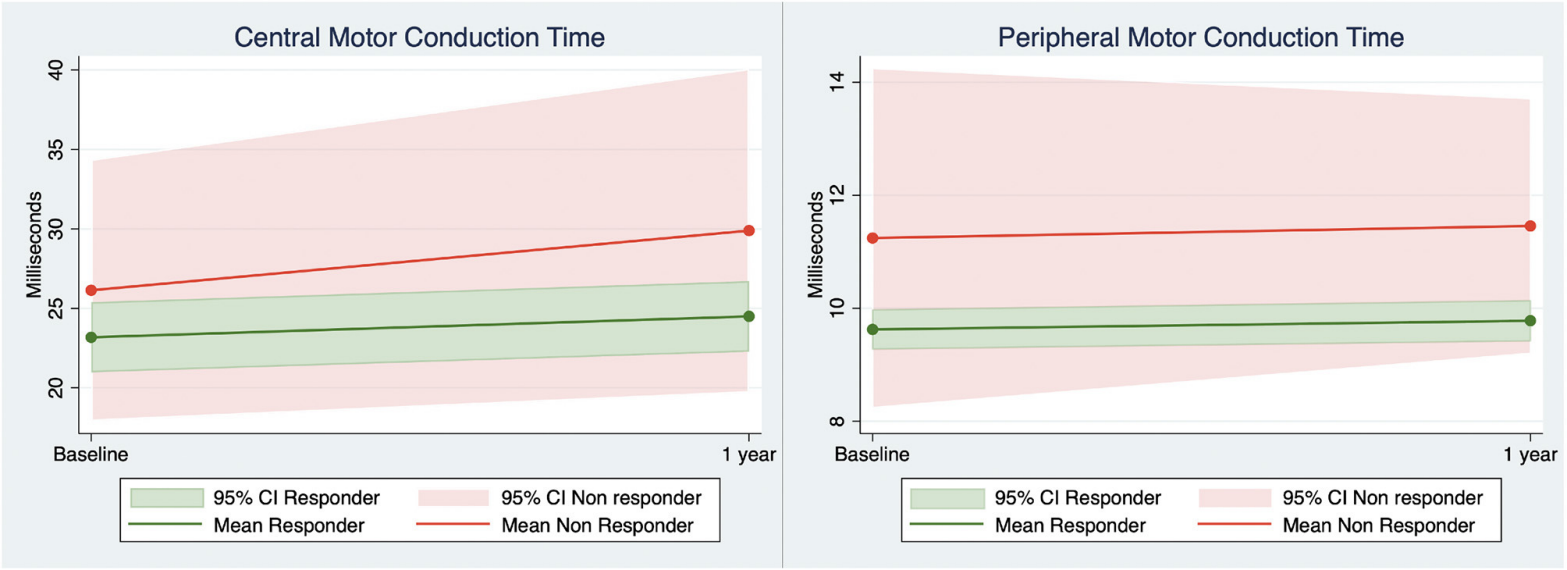
Central and peripheral motor conduction (vastus medialis muscle) derived from transcranial magnetic stimulation elicited motor evoked potentials in responders and non-responders to Fampridine treatment at baseline and 1-year follow-up. 95% CI, 95% confidence interval.

At baseline and follow-up, there were no statistically significant intergroup differences in CMCT, while the mean CMCT of non-responders was above the upper 95% CI of responders to Fampridine treatment at both visits (Figure 3). There were no intergroup differences in PMCT in non-responders compared to responders when examined at baseline and follow-up (*p* > 0.05, Table 2). However, when analyzing the PMCT across time points and adjusting for response status, age, and disease duration in the mixed effect regression models, non-responders had significantly prolonged PMCT when compared to responders to Fampridine treatment (*p* < 0.006, **Table 4**). There were no intergroup differences at baseline and follow-up in MEP amplitudes and ENG measures (*p* > 0.05).

**Table 2:** Neurophysiological examinations at baseline and 1 year in responders and non-responders to Fampridine treatment.

	**Responders**				**Non-responders**				**Intergroup difference**	
	**Baseline**	**Follow-up**	**Mean and**	***p*-Value**	**Baseline**	**Follow-up**	**Mean and**	***p*-Value**	**Baseline**	**Follow-up**
	**(*n* = 41)**	**(*n* = 39)**	**%-change**		**(*n* = 8)**	**(*n* = 8)**	**%-change**		***p*-value**	***p*-value**
CMCT; *ms*	23.2 ± 7.0	24.5 ± 6.9	1.5 ± 2.2 (8.0 ± 11.0%)	** <0.001** ^**a**^	26.1 ± 9.9	29.9 ± 12.2	3.8 ± 4.1 (13.5 ± 15.9%)	**0.035** ^**a**^	0.498^d^	0.087^c^
Amplitude; mV										
Cortex – Vastus medialis	0.9 ± 0.7	0.4 ± 0.5	−0.4 ± 0.6 (−43.7 ± 37.6%)	** <0.001** ^**b**^	0.9 ± 0.7	0.3 ± 0.2	−0.6 ± 0.7 (−57.9 ± 26.3%)	**0.008** ^**b**^	0.735^d^	0.288^d^
Cortex – Tibialis anterior	1.6 ± 1.4	1.0 ± 1.1	−0.7 ± 0.9 (−35.0 ± 48.0%)	** <0.001** ^**b**^	1.0 ± 0.7	0.4 ± 0.1	−0.5 ± 0.6 (−49.4 ± 31.6%)	**0.032** ^**a**^	0.082^e^	0.126^d^
PMCT; *ms*										
Vastus medialis	9.6 ± 1.1	9.8 ± 1.1	0.2 ± 1.2 (2.7 ± 13.0%)	0.270^a^	11.2 ± 3.6	11.5 ± 2.7	0.2 ± 2.3 (5.3 ± 18.3%)	0.797^**a**^	0.245^e^	0.124^e^
Tibialis anterior	15.2 ± 1.4	15.9 ± 1.5	0.7 ± 1.2 (4.9 ± 8.2%)	** <0.001** ^**b**^	18.0 ± 6.0	17.8 ± 3.5	−0.1 ± 3.8 (2.7 ± 14.4%)	0.195^**b**^	0.228^e^	0.162^e^
Amplitude; *mV*										
Spine – Vastus medialis	1.8 ± 1.8	0.7 ± 0.6	−1.1 ± 1.5 (−44.6 ± 47.4%)	** <0.001** ^**b**^	0.8 ± 0.6	0.6 ± 0.5	−0.2 ±.04 (−21.8 ± 46.3%)	0.173^**a**^	0.083^d^	0.901^d^
Spine – Tibialis anterior	0.8 ± 0.5	0.6 ± 0.5	−0.2 ± 0.8 (−47.8 ± 85.6%)	<0.170^a^	0.8 ± 0.6	0.4 ± 0.3	−0.4 ± 0.7 (−13.1 ± 89.1%)	0.161^**a**^	0.978^d^	0.436^d^
Latency; *ms*										
Peroneal nerve	4.5 ± 0.7^*^	4.4 ± 0.8^*^	−0.1 ± 0.7 (-1.1 ± 14.2%)	0.496^a^	4.6 ± 0.7^*^	4.8 ± 1.2^*^	0.2 ± 0.8 (4.3 ± 19.5%)	0.552^a^	0.787^c^	0.306^c^
Tibial nerve	4.0 ± 0.6	3.8 ± 0.7	−0.1 ± 0.6 (−2.8 ± 15.7%)	0.168^a^	3.7 ± 0.7	3.7 ± 0.6	−0.1 ± 0.4 (−1.0 ± 11.8%)	0.623^a^	0.245^c^	0.151^d^
Amplitude; *mV*										
Peroneal nerve	6.4 ± 2.6^*^	6.3 ± 2.6^*^	−0.2 ± 1.7 (3.2 ± 31.7%)	0.565^a^	5.2 ± 1.0^*^	11.5 ± 2.7^*^	1.0 ± 3.2 (27.0 ± 77.5%)	0.813^b^	**0.046** ^**e**^	0.707^d^
Tibial nerve	15.0 ± 6.7	16.2 ± 5.5	1.2 ± 4.9 (26.4 ± 76.7%)	0.190^b^	13.8 ± 10.2	14.7 ± 10.2	0.9 ± 3.8 (28.5 ± 91.5%)	0.429^a^	0.695^c^	0.569^d^
CV; *m/s*										
Peroneal nerve	46.2 ± 5.8^*^	45.8 ± 3.7^*^	−0.5 ± 5.2 (0.1 ± 9.9%)	0.964^b^	44.7 ± 6.3^*^	44.5 ± 5.1^*^	−0.2 ± 2.8 (0.0 ± 6.3%)	0.527^a^	0.622^d^	0.627^d^
Tibial nerve	43.1 ± 5.0	43.7 ± 4.1	0.4 ± 4.2 (1.7 ± 10.5%)	0.543^a^	39.8 ± 3.7	42.3 ± 4.4	2.5 ± 2.9 (6.5 ± 7.7%)	**0.008** ^**b**^	0.083^c^	0.377^c^
F-waves; *ms*										
Peroneal nerve	53.0 ± 5.3^*^	53.1 ± 5.2^*^	0.3 ± 2.5 (0.7 ± 4.6%)	0.454^a^	54.4 ± 5.7^*^	56.0 ± 3.5^*^	1.7 ± 4.8 (3.7 ± 9.7%)	0.396^a^	0.526^c^	0.167^c^
Tibial nerve	53.3 ± 4.8	53.9 ± 5.6	0.8 ± 3.6 (1.6 ± 6.6%)	0.491^b^	55.5 ± 5.1	57.0 ± 4.8	1.5 ± 2.2 (2.8 ± 4.2%)	0.095^a^	0.244^c^	0.150^c^

*p-values derived from*

a *paired t-tests*,

b *Wilcoxon matched pairs signed rank test*,

c *unpaired t-tests*,

d *Wilcoxon rank-sum test, and*

e *Welch test due to significant Levene's test (p < 0.05) suggesting unequal variance*.

* *in responders n = 37 at baseline, n = 35 (follow-up), and non-responders n = 7. Bold p-values signify statistically significant differences. CMCT, central motor conduction time; PMCT, peripheral motor conduction time; ms, milliseconds; mV, millivolts; m/s, meters per second*.

After 1 year (Table 2), both groups had significant CMCT prolongation and a decrease in cortical MEP amplitudes (*p* < 0.035). Four responders were not able to undergo ENG of the right peroneal nerve due to atrophied extensor digitorum brevis muscles and the same applied to one non-responder and the left peroneal nerve.

### Magnetic Resonance Imaging

At baseline and follow-up, there were no intergroup differences in brain volume, number and volume of T2-weighted lesions, and lesion load normalized to brain volume (*p* > 0.05). At follow-up (Table 3), brain volume was decreased (*p* < 0.001) and lesion load normalized to brain volume increased in responders to Fampridine treatment (*p* = 0.007).

**Table 3:** Changes in magnetic resonance imaging outcomes at baseline and after 1 year in responders and non-responders to Fampridine treatment.

	**Responders**				**Non-responders**				**Intergroup difference**	
	**Baseline**	**Follow-up**	**Mean and**	***p*-Value**	**Baseline**	**Follow-up**	**Mean and**	***p*-Value**	**Baseline**	**Follow-up**
	**(*n* = 41)**	**(*n* = 39)**	**%-change**		**(*n* = 8)**	**(*n* = 8)**	**%-change**		***p*-value**	***p*-value**
Brain volume *ml*	1,377 ± 147	1,363 ± 142	−7.3 ± 11.6 (−0.5 ± 0.8%)	** <0.001** ^**b**^	1,474 ± 131	1,461 ± 134	−13.8 ± 24.4 (−0.9 ± 1.7%)	0.078^b^	0.092^c^	0.080^c^
Number of T2-weighted lesions	19.2 ± 10.0	19.8 ± 10.5	0.3 ± 1.8 (1.5 ± 12.8%)	0.408^b^	18.1 ± 6.2	18.3 ± 5.8	0.1 ± 1.1 (1.9 ± 7.1)	0.763^a^	0.735^d^	0.571^d^
Volume of T2-weighted lesions (lesion load); *ml*	8.9 ± 12.6	9.3 ± 13.0	0.2 ± 1.1 (3.4 ± 18.2%)	0.079^b^	10.3 ± 9.5	10.0 ± 9.8	−0.3 ± 0.8 (−3.4 ± 7.3%)	0.374^a^	0.208^d^	0.240^d^
Lesion load normalized to brain volume; ‰	14.0 ± 7.1	14.6 ± 7.6	0.3 ± 1.4 (2.0 ± 12.9%)	**0.007** ^**b**^	12.3 ± 4.4	12.6 ± 4.3	0.3 ± 0.8 (2.9 ± 6.9%)	0.326^a^	0.527^c^	0.485^c^

*p-values derived from*

a *paired t-tests*,

b *Wilcoxon matched pairs signed rank test*,

c *unpaired t-tests, and*

d *Wilcoxon rank-sum test. Bold p-values signify statistically significant differences. ml, milliliters*.

### Associations Between Disability, Physical Performance Tests, and Neurophysiology

Mixed effect regression models including changes over time in the entire study population demonstrated that the EDSS was associated with the CMCT in the fully adjusted analysis (*p* = 0.031). Univariate and partly adjusted analyses showed associations between MSWS-12 and T25FW (*p* = 0.026), SSST (*p* = 0.021), and CMCT (*p* < 0.0001), where only the T25FW (*p* = 0.040) and CMCT (*p* = 0.001) were associated in the fully adjusted model.

### Central and Peripheral Motor Conduction Times, Walking Performance, and Brain Volume

Mixed effect regression analysis, including changes over time, demonstrated that CMCT in the entire study population was associated with only the SSST in the univariate and partly adjusted analysis (*p* = 0.029; Table 4). Coefficients from the mixed-effect regression model showed that SSST increased by 1 s when the CMCT increased by 0.11 ms (Table 4). Peripheral motor conduction times of the VM muscle were not associated with any walking test or brain volume.

**Table 4:** Linear mixed regression models and associations between disability, physical and cognitive performance, neurophysiology, and MRI.

		**Univariate**		**Partly adjusted**		**Fully adjusted**	
**Outcome**	**Variable**	**Coefficient** **(95% CI)**	***p*-Value**	**Coefficient (95 % CI)**	***p*-Value**	**Coefficient** **(95% CI)**	***p*-Value**
**EDSS**	Responder	0.38 (−0.47; 1.42)	0.325	0.48 (−0.47; 1.42)	0.325	0.38 (−0.52; 1.29)	0.406
	T25FW	0.03 (−0.01; 0.04)	0.163	0.02 (−0.01; 0.04)	0.163	−0.02 (−0.07; 0.04)	0.587
	SSST	0.02 (0.00; 0.02)	0.086	0.01 (0.00; 0.02)	0.086	0.02 (−0.02; 0.05)	0.297
	CMCT	0.03 (0.00; 0.05)	0,057	0.03 (0.00; 0.05)	0.057	0.03 (0.00; 0.06)	**0.031**
	PMCT VM	−0.02 (−0.09; 0.03)	0.321	−0.03 (−0.09; 0.03)	0,321	−0.03 (−0.09; 0.04)	0,395
	Brain volume	0.00 (0.00; 0.00)	0.420	0.00 (0.00; 0.00)	0.420	0.00 (0.00; 0.00)	0.379
**MSWS-12**	Responder	−1.06 (−7.41; 5.92)	0.826	−0.75 (−7.41; 5.92)	0.826	1.39 (−4.7; 7.49)	0.654
	T25FW	0.25 (0.03; 0.46)	**0.026**	0.24 (0.03; 0.46)	**0.026**	1.02 (0.05; 2)	**0.040**
	SSST	0.14 (0.02; 0.25)	**0.021**	0.13 (0.02; 0.25)	**0.021**	−0.43 (−0.93; 0.07)	0.090
	CMCT	0.45 (0.23; 0.77)	**<0.0001**	0.5 (0.23; 0.77)	**<0.0001**	0.47 (0.2; 0.74)	**0.001**
	PMCT VM	−0.72 (−1.7; 0.31)	0.177	−0.69 (−1.7; 0.31)	0.177	−0.51 (−1.6; 0.58)	0.361
	Brain volume	0.01 (−0.01; 0.03)	0.245	0.01 (−0.01; 0.03)	0.245	0.01 (−0.01; 0.02)	0.294
**CMCT**	Responder	−4.12 (−9.24; 1.93)	0.200	−3.65 (−9.24; 1.93)	0.200	−2.67 (−8.33; 3)	0.356
	T25FW	0.18 (−0.01; 0.32)	0.072	0.15 (−0.01; 0.32)	0.072	0.04 (−0.42; 0.51)	0.854
	SSST	0.11 (0.01; 0.19)	**0.029**	0.10 (0.01; 0.19)	**0.029**	0.07 (−0.2; 0.34)	0.592
	Brain volume	0.00 (−0.01; 0.02)	0.286	0.01 (−0.01; 0.02)	0.286	0.00 (−0.01; 0.02)	0.532
**PMCT VM**	Responder	−1.64 (−2.74; −0.62)	**0.002**	−1.68 (−2.74; −0.62)	**0.002**	−1.55 (−2.65; −0.44)	**0.006**
	T25FW	−0.02(−0.06; 0.02)	0.244	−0.02 (−0.06; 0.02)	0.244	0.00 (−0.18; 0.19)	0.971
	SSST	−0.01 (−0.04; 0.01)	0.229	−0.01 (−0.04; 0.01)	0.229	−0.02 (−0.11; 0.08)	0.720
**SDMT**	Responder	1.92 (−4.38; 7.81)	0.582	1.71 (−4.38; 7.81)	0.582	0.00 (−6.63; 6.64)	0.999
	Brian volume	−0.01 (−0.02; 0.01)	0.776	0.00 (−0.02; 0.01)	0.776	−0.02 (−0.06; 0.02)	0.380
	No. T2-weighted lesions	−0.41 (−0.56; −0.03)	**0.026**	−0.30 (−0.56; −0.03)	**0.026**	0.91 (−1.41; 3.23)	0.443
	Volume of T2-weighted lesions	−0.28 (−0.43; −0.03)	**0.026**	−0.23 (−0.43; −0.03)	**0.026**	−0.15 (−0.37; 0.07)	0.172
	Lesion load normalized to brain volume	−0.54 (−0.77; −0.06)	**0.023**	−0.42 (−0.77; −0.06)	**0.023**	−1.55 (−4.73; 1.63)	0.340

*95% CI, 95% confidence interval; EDSS, expanded disability status scale; CMCT, central motor conduction time; PMCT, peripheral motor conduction time; VM, vastus medialis; MSWS-12, 12-item multiple sclerosis walking scale; T25FW, timed 25-foot walk test; SSST, six spot step test; 5-STS, 5-times sit-to-stand test; SDMT, symbol digit modalities test. The bold p-values signify statistically significant associations between outcome and variable*.

### Symbol Digit Modalities Test and MRI Outcomes

Univariate and partly adjusted analysis of the entire study population (Table 4) showed that the SDMT was associated with number of T2-weighted lesions, lesion load, and lesion load normalized to brain volume (0.023 < *p* < 0.026). Coefficients from the mixed-effect regression showed that an increase of 1 MS lesion on MRI is associated with a decrease of 0.41 arbitrary units on the SDMT (Table 4).

## Discussion

This longitudinal study demonstrated that non-responders to Fampridine had prolongation of PMCT throughout the study when compared to responders. We did not find intergroup differences in disability measures, physical and cognitive performance tests, CMCT, ENG, and MRI outcomes.

In the entire study population, EDSS was associated with CMCT, whereas the self-reported MSWS-12 was associated with T25FW, SSST, and CMCT. The CMCT was also associated with the SSST. The SDMT was associated with number of T2-weighted lesions, lesion load, and lesion load normalized to brain volume.

### Central and Peripheral Nerve Conduction and Fampridine Responsiveness

We observed a trend toward prolonged CMCT in non-responders compared to responders to Fampridine treatment after 1 year (*p* = 0.087), at a level exceeding the upper limit of the 95% CI of responders (Figure 3). The lack of statistical significance is most likely a type II error caused by the low number of non-responders. Cortical MEP amplitudes decreased at 1-year follow-up suggesting CNS neuroaxonal damage in both groups (35). There was no clear pattern of neuroaxonal damage in the PNS as measured by ENG amplitudes and spinal MEP amplitudes. The pattern of demyelination found in the spinal MEP latencies, therefore suggests that the proximal part of the PNS in non-responders to Fampridine treatment is involved in MS disease mechanisms, as of yet not elucidated disease mechanism. Currently, the exact mechanism of Fampridine in the CNS and PNS is not understood as a definite molecular target has not been identified and the repertoire of potassium channels, on which it exerts its effect, are not completely understood (1, 36). In a study by Leussink et al. on patients with inflammatory demyelination of the PNS, Fampridine was ineffective in restoring nerve conduction (36). Furthermore, it was suggested that the lack of Fampridine response in the PNS may be a result of different tissue distribution or degree of demyelination (36). The latter may be supported by our finding of prolonged PMCT in the non-responders to Fampridine treatment which suggest a higher degree of demyelination in the proximal part of the PNS, compared to responders. The affection of the PNS in PwMS has also been demonstrated in a magnetic resonance neurography study by Jende et al., demonstrating a higher number of T2-weighted lesions in the sciatic nerve and higher tibial and peroneal nerve calibers in the PwMS compared to healthy controls, with simultaneous normal ENG findings in PwMS (37). Other studies have suggested the role of neuroaxonal damage (38), Wallerian degeneration involving longer fascicular nerve segments (38), and epitope spreading of demyelinating peripheral neuropathy (39) as neurodegenerative components of disease progression in MS.

Taken together, our results support that Fampridine improves nerve conduction in the CNS and PNS in responders. The involvement of the PNS as a part of the disease mechanism in MS may contribute to Fampridine non-responsiveness, and future studies should therefore further evaluate PMCT as a potential biomarker of Fampridine responsiveness.

### Walking Tests

Unlike the T25FW, the SSST reflected nerve conduction in the CNS, as it was associated with CMCT. In earlier studies, the SSST has been demonstrated to be more responsive to Fampridine treatment (13) with higher sensitivity and discriminatory power than the T25FW (24). In a study on patients with chronic inflammatory polyneuropathy, SSST has been suggested to be an alternative test for monitoring walking due to its superior dynamic range, floor effect, and responsiveness when compared to T25FW (40). This finding adds support to the implementation of the SSST as a routine walking test in monitoring PwMS.

### Central Motor Conduction Times and Measures of Disability

Central motor conduction time was associated with EDSS in the fully adjusted analysis and MSWS-12 in all mixed linear regression models. The self-reported MSWS-12 has previously been shown as a strong predictor of EDSS (41). In addition, studies have shown a strong relationship between baseline evoked potentials and future disability measured by EDSS, especially in the early stages of relapsing–remitting and primary progressive MS (42, 43). When MS lesions affect corticospinal tracts, MEPs are proficient in detecting clinical and subclinical nerve conduction alterations, especially in the lower extremities (44).

To our knowledge, this is the first study to examine the association between CMCT and the self-reported MSWS-12 over 1 year. In a study examining changes in walking impaired PwMS treated with Fampridine over a 2-week trial, Brambilla et al. also demonstrated a significant correlation between CMCT and MSWS-12 (20). The strong association of MSWS-12 with CMCT and walking tests observed in the present study, therefore, adds further support to the use of the self-reported MSWS-12 in monitoring disability in PwMS.

### MRI Outcomes and SDMT

There were no intergroup differences regarding SDMT and MRI outcomes. After 1 year brain volume (Table 3) decreased in responders to Fampridine treatment (mean −0.5%, *p* < 0.001) and non-responders (−0.9%, *p* = 0.078) which is more than in healthy people (−0.04%/year and a loss of 0–0.5% according to meta-analyses) (45). Loss of brain volume is considered to be a reflection of neuroaxonal damage and demyelination (46, 47), which is also somewhat supported by the significant reduction of cortical MEP amplitudes in both groups. Of note, however, brain volume was not associated with disability measures (EDSS and MSWS-12) in this study.

In a study applying DTI, before and after a 2-week trial with Fampridine, Brambilla et al. found that responders had a significant reduction in mean and radial diffusivity in corticospinal tracts compared to non-responders, which was assumed to be caused by the closure of potassium channels and modification of osmotic balance of water molecules across axonal membranes (20). In another DTI study, Klineova et al. suggested corticospinal tract MD as a main candidate biomarker to predict Fampridine responsiveness (48).

Both responders and non-responders deteriorated in the SDMT at 1-year follow-up. In the entire study population, SDMT was associated with number and volume of T2-weighted lesions, and lesion load normalized to brain volume. Studies have also shown that cognitive processing speed is associated with lesion load (49). Due to stronger associations with MRI outcomes, it has been suggested that the SDMT is suited to replace the Paced Auditory Serial Addition Test in the MS Functional Composite (50).

## Limitations

The study was designed as an explorative study to evaluate potential biomarkers that could aid in future studies regarding the identification of PwMS who will respond to Fampridine treatment. The main limitation is the small number of non-responders to Fampridine treatment increasing the risk of type II errors. It may be speculated whether the initially significant exposure variables in univariate and partly adjusted analyses, which turn out insignificant in the fully adjusted analysis, are due to multicollinearity or confounding factors. Also, examinations were performed unblinded, which could introduce bias.

Participants were already established regarding their response to Fampridine prior to inclusion, which could challenge the interpretation of results, as baseline outcome measures prior to initiation of treatment with Fampridine may have been different.

Finally, different MRI scanners and sequences were utilized as the study was multicentered. Scanners may have different sensitivities in detecting T2-weighted lesions and otherwise affect image-based outcomes. Furthermore, the lack of MRI of the spine can also present challenges in interpreting results as lesions in the spinal cord also contribute to neurophysiological and clinical changes.

## Clinical Implications

Our results demonstrate that the PMCT may be used as a marker for Fampridine responsiveness in PwMS. Our results support the usefulness of the MSWS-12 in monitoring disease progression of PwMS in the clinical setting, as it is strongly associated with the CMCT and walking tests. Furthermore, CMCT can be utilized as a marker of disability progression in MS, while the SDMT can be used as a marker identifying accumulation of number and volume of MS lesions visualized on MRI of the brain.

## Conclusion

In this 1-year longitudinal study, non-responders to Fampridine treatment had prolonged PMCT when compared to responders. Peripheral motor conduction times may be a useful biomarker associated with Fampridine response. There were no intergroup differences in disability measures, other physical and cognitive performance tests, CMCT, ENG, or MRI outcomes between responders and non-responders to Fampridine treatment.

The CMCT was associated with both the SSST and the MSWS-12 emphasizing its potential clinical usefulness.

## Data Availability Statement

The raw data supporting the conclusions of this article will be made available by the authors, without undue reservation.

## Ethics Statement

The studies involving human participants were reviewed and approved by The National Committee on Health Research Ethics (Denmark). Project identification: S-20160204. The patients/participants provided their written informed consent to participate in this study.

## Author Contributions

All authors listed have made a substantial, direct and intellectual contribution to the work, and approved it for publication.

## Funding

This study was funded by the Region of Southern Denmark, University of Southern Denmark, University Hospital of Southern Jutland, Knud and Edith Eriksens Mindefond, and the charity-event of Rotary Denmark Night of a 1000 drawings in Esbjerg.

## Conflict of Interest

UD has received research support, travel grants, and/or teaching honorary from Biogen Idec, Merck Serono, Novartis, Bayer Schering, and Sanofi Aventis as well as honoraria from serving on scientific advisory boards of Biogen Idec and Genzyme. The remaining authors declare that the research was conducted in the absence of any commercial or financial relationships that could be construed as a potential conflict of interest.

## Publisher's Note

All claims expressed in this article are solely those of the authors and do not necessarily represent those of their affiliated organizations, or those of the publisher, the editors and the reviewers. Any product that may be evaluated in this article, or claim that may be made by its manufacturer, is not guaranteed or endorsed by the publisher.
